# Editorial: Antibiotic Resistance in Aquatic Systems

**DOI:** 10.3389/fmicb.2017.00014

**Published:** 2017-01-25

**Authors:** Satoru Suzuki, Amy Pruden, Marko Virta, Tong Zhang

**Affiliations:** ^1^Center for Marine Environmental Studies, Ehime UniversityMatsuyama, Japan; ^2^Department of Civil and Environmental Engineering, Virginia TechBlacksburg, VA, USA; ^3^Department of Food and Environmental Sciences, University of HelsinkiHelsinki, Finland; ^4^Department of Civil Engineering, The University of Hong KongHong Kong, Hong Kong

**Keywords:** antibiotic resistance, aquatic systems, wastewater treatment plant, aquaculture, horizontal gene transfer

The spread of antibiotic-resistant pathogens and their resistance traits is an epic global challenge, as recognized by various international bodies, including the G8 Science Ministry in 2013 and the Elmau summit in 2015 (https://www.g7germany.de/Webs/G7/EN/Home_en/home_node.html). While most attention continues to be devoted to the clinic and the need to develop new drugs, there is growing recognition of the need to understand the origin and ecology of antibiotic resistance in order to slow its spread and maximize the lifespan of our antibiotic arsenal (Wright, 2010). In particular, the aquatic environment can serve both as a natural reservoir of antibiotic resistance and a conduit for the spread of clinical resistance traits of major concern (Michael et al., 2013). Aquatic bodies, including lakes, rivers, streams and even coastlines, receive effluent from wastewater treatment plants (WWTP), runoff from agricultural activity, and other human inputs and influences that may either serve to elevate natural background levels of antibiotic resistance genes (ARGs) and stimulate their transfer into pathogens or other organisms, or as a conduit for the propagation of antibiotic resistant pathogens and clinical ARGs of concern (Michael et al., 2013). Current pathogen risk models are not equipped to factor in unique challenges that antibiotic resistance poses, including the potential that non-pathogenic antibiotic resistant bacteria (ARB) can serve as a reservoir to transfer their ARGs to pathogens or the role of selective agents, such as antibiotics and metals, in amplifying this potential. The challenge of controlling the spread of antibiotic resistance can have a different face in developed or developing countries, depending on local policies, practices, technologies and constraints.

The articles in this e-book include new evidences of the origin, spread, and fate of ARB and ARGs in aquatic systems, focusing on water systems from wastewater, freshwater to seawater. These various media have gained attention as potential sources, sinks, or conduits in the potential to spread antibiotic resistance.

It is well-known that heavy use of antibiotics and synthetic antimicrobial agents contribute to the selection pressure. The fate and impact of antibiotics used both in humans and livestock are of particular concern. Municipal and on-farm wastewater treatment is critical for controlling pollution and pathogens, but is not tailored specifically to the control of antibiotic resistance. WWTPs are mainly aimed at reducing solid and nutrient loads to surface waters, but this does not guarantee biodegradation of trace chemical pollutants or genetic elements. Although advanced disinfection facilities can greatly reduce the danger of waterborne diseases (United States Environmental Protection and Agency, 2004), antibiotics and ARGs can still be released to the environment in disinfected effluents (Michael et al., 2013; Rizzo et al., 2013; Berkner et al., 2014; Carey and McNamara). Recent works report the improvement of disinfection in terms of ARG removal (Munir et al., 2011; McKinney and Pruden, 2012; Guo et al., 2013; Yuan et al., 2015; Zhuang et al., 2015).

The potential for horizontal gene transfer (HGT) in WWTPs is a matter of importance, and there is debate regarding the favorable conditions and the actual HGT rates. In this e-book, Miller et al. explore the extent to which influent ARB and ARG composition in raw sludge influences the fate of ARB in the digested sludge community and potential for ARG transfer. Enhancement of HGT by the ionic liquid (IL) 1-butyl-3-methylimidazolium hexafluorophosphate showed that longer carbon chain enhanced HGT (Wang et al.). IL has been thought as an environmentally-friendly solvent, but recently water solubility, environmental toxicity and stability of IL are considered to be risk (Pham et al., 2010). The enhancement of HGT is a new effect of IL. Vanadium is also reported to have enhancement effect of HGT in environment (Suzuki et al., 2012). Such studies demonstrate that, besides antibiotics, other chemicals enhancing HGT could play a role to spread ARGs in water environments, including between pathogenic bacteria and environmental bacteria. In case of coastal sea, although the contaminated waters are significantly diluted, fecal-derived bacteria, such as *E. coli*, remain viable ARG reservoirs of concern (Alves et al.; Kappell et al.; Ghaderpour et al.). ARB and ARGs are known to occur in aquatic environments without antibiotic contaminations (Port et al., 2014). ARBs and ARGs flow into rivers, groundwater and marine environments by influx of WWTP effluent as mentioned above. Additionally, there is marked potential aquaculture practices (Tamminen et al., 2011) and open ocean conditions (Rahman et al., 2008) to contribute to the selection and spread of ARBs and ARGs. Mobile genetic elements (MGEs) of aquatic species conveying multidrug resistance genes are reported in this book (Nonaka et al.). These elements might pose a risk to human health if the MGEs transfer to other bacteria (Piotrowska and Popowska), especially human pathogens. Thus, this topic highlights insight obtained from various water environments and their interfaces.

The role of antibiotics and antimicrobials in ARB selection is of keen interest and evidence suggests that even very low concentration of antibiotics can be effective to select and maintain resistance traits in bacteria (Gullberg et al., 2011). Marine bacterial plasmids pAQUs (Nonaka et al.) appear to disseminate ARGs among marine organisms, which can be stably retained in the bacterial community, even after the antibiotics are removed (Bien et al., 2015). Exogenous ARGs retained in the environment can potentially be horizontally transferred among the native bacterial community.

It is well-known that the majority of aquatic environmental bacteria are unculturable or yet-to-be cultured (Amann et al., 1995; Takami et al., 1997; Bloomfield et al., 1998). This characteristic is distinct from that of human and animal pathogens, which have been the subject of the development of standard methods for isolation and monitoring. Consequently, conventional culture-dependent methods for monitoring ARBs and ARGs only reveal 0.1% or less of the true aquatic bacterial community (Amann et al., 1995; Takami et al., 1997; Bloomfield et al., 1998). Suzuki et al. (2013) and Suzuki et al. found that the defining features of ARGs tend to be distinct between culturable and yet-to-be cultured bacteria. The recent advent of next-generation DNA sequencing and metagenomics poises the scientific community on the verge of a major advance in understanding the behavior of ARGs in the environment (D'Costa et al., 2006; Port et al., 2014). However, deep sequencing and the capacity to perform high-throughput bioinformatics analyses are required to match ARGs with their bacterial hosts. Thus, there is still a long way to go before we have full understanding of the factors driving the behavior of ARGs in aquatic realm.

Articles in this e-book provide new insight into the dynamics of ARGs in a diverse range of aquatic environments and their interfaces, including distribution and diversity of ARGs in various countries, horizontal gene transfer, and the dynamic processes involved in governing ARB and ARG fate. These papers should expand our knowledge and understanding of connections between resistance traits in human- and animal-related bacteria and aquatic ecosystems.

Although this e-book does not include, gene flux among various aquatic compartments, such as sediments, biofilms and periphyton, should be paid attention. ARGs are exchanged between organisms, but it does not drive mass movement and local exposures. As an example, movement of ARGs is reported between water column and biofilms (Engemann et al., 2008). The geological wide flux of ARGs is occurred by natural water movement (Knapp et al., 2012), and water use and food are suspected also as ARGs transport factors (Suzuki and Hoa, 2012). The study from global ecological viewpoint is needed to reveal the ARGs fate and dynamics in environments.

## Author contributions

SS: Planning, manuscript editing and making editorial manuscript. AP: Manuscript editing and making editorial manuscript. MV: Manuscript editing. TZ: Manuscript editing and reviewing editorial manuscript.

## Funding

This work is partly supported by Grant-in-Aid KAKENHI 25257402 and 22241014.

### Conflict of interest statement

The authors declare that the research was conducted in the absence of any commercial or financial relationships that could be construed as a potential conflict of interest.
